# Supplementary material for: Clozapine and Pneumonia: Synthesizing the Link by Reviewing Existing Reports—A Systematic Review and Meta-Analysis
Source: Medicina (Kaunas). 2024 Dec 6;60(12):2016. doi: 10.3390/medicina60122016 (PMC11728434; doi:10.3390/medicina60122016)
Supplement: Supplementary file 1 [file medicina-60-02016-s001.zip › medicina-3333516-supplementary.pdf]

Supplementary tables

Table S1

PRISMA 2020 Main Checklist

Topic	No.	Item	Location where item is reported
**TITLE**			
**Title**	1	Identify the report as a systematic review.	Main Title, Line 1
**ABSTRACT**			
**Abstract**	2	See the PRISMA 2020 for Abstracts checklist	
**INTRODUCTION**			
**Rationale**	3	Describe the rationale for the review in the context of existing knowledge.	Line 59-75
**Objectives**	4	Provide an explicit statement of the objective(s) or question(s) the review addresses.	Line 77-80
**METHODS**			
**Eligibility criteria**	5	Specify the inclusion and exclusion criteria for the review and how studies were grouped for the syntheses.	Table 2
**Information sources**	6	Specify all databases, registers, websites, organizations, reference lists and other sources searched or consulted to identify studies. Specify the date when each source was last searched or consulted.	Line 84, Supplementary Table S2
**Search strategy**	7	Present the full search strategies for all databases, registers and websites, including any filters and limits used.	Supplementary Table S2
**Selection process**	8	Specify the methods used to decide whether a study met the inclusion criteria of the review, including how many reviewers screened each record and each report retrieved, whether they worked independently, and if applicable, details of automation tools used in the process.	Line 88-100, Table 1

Topic	No.	Item	Location where item is reported
**Data collection process**	9	Specify the methods used to collect data from reports, including how many reviewers collected data from each report, whether they worked independently, any processes for obtaining or confirming data from study investigators, and if applicable, details of automation tools used in the process.	Line 121-128
**Data items**	10a	List and define all outcomes for which data were sought. Specify whether all results that were compatible with each outcome domain in each study were sought (e.g. for all measures, time points, analyses), and if not, the methods used to decide which results to collect.	Table 2
	10b	List and define all other variables for which data were sought (e.g. participant and intervention characteristics, funding sources). Describe any assumptions made about any missing or unclear information.	No additional variables
**Study risk of bias assessment**	11	Specify the methods used to assess risk of bias in the included studies, including details of the tool(s) used, how many reviewers assessed each study and whether they worked independently, and if applicable, details of automation tools used in the process.	Line 139-142
**Effect measures**	12	Specify for each outcome the effect measure(s) (e.g. risk ratio, mean difference) used in the synthesis or presentation of results.	Line 122-123
**Synthesis methods**	13a	Describe the processes used to decide which studies were eligible for each synthesis (e.g. tabulating the study intervention characteristics and comparing against the planned groups for each synthesis (item 5)).	Line 124-126
	13b	Describe any methods required to prepare the data for presentation or synthesis, such as handling of missing summary statistics, or data conversions.	Line 123-124
	13c	Describe any methods used to tabulate or visually display results of individual studies and syntheses.	Line 129-130
	13d	Describe any methods used to synthesize results and provide a rationale for the choice(s). If meta-analysis was performed, describe the model(s), method(s) to identify the presence and extent of statistical heterogeneity, and software package(s) used.	Line 129-130
	13e	Describe any methods used to explore possible causes of heterogeneity among study results (e.g. subgroup analysis, meta-regression).	Line 135-136
	13f	Describe any sensitivity analyses conducted to assess robustness of the synthesized results.	Line 140-142
**Reporting bias assessment**	14	Describe any methods used to assess risk of bias due to missing results in a synthesis (arising from reporting biases).	Line 135-140

Topic	No.	Item	Location where item is reported
**Certainty assessment**	15	Describe any methods used to assess certainty (or confidence) in the body of evidence for an outcome.	N/A
**RESULTS**			
**Study selection**	16a	Describe the results of the search and selection process, from the number of records identified in the search to the number of studies included in the review, ideally using a flow diagram.	Figure 1
	16b	Cite studies that might appear to meet the inclusion criteria, but which were excluded, and explain why they were excluded.	N/A
**Study characteristics**	17	Cite each included study and present its characteristics.	Table 5
**Risk of bias in studies**	18	Present assessments of risk of bias for each included study.	Figure 4 and 5
**Results of individual studies**	19	For all outcomes, present, for each study: (a) summary statistics for each group (where appropriate) and (b) an effect estimate and its precision (e.g. confidence/credible interval), ideally using structured tables or plots.	Figure 2 and 3
**Results of syntheses**	20a	For each synthesis, briefly summarize the characteristics and risk of bias among contributing studies.	Line 181-191
	20b	Present results of all statistical syntheses conducted. If meta-analysis was done, present for each the summary estimate and its precision (e.g. confidence/credible interval) and measures of statistical heterogeneity. If comparing groups, describe the direction of the effect.	Line 426-433
	20c	Present results of all investigations of possible causes of heterogeneity among study results.	Line 403-433
	20d	Present results of all sensitivity analyses conducted to assess the robustness of the synthesized results.	Figure 4 and 5
**Reporting biases**	21	Present assessments of risk of bias due to missing results (arising from reporting biases) for each synthesis assessed.	Figure 4 and 5
**Certainty of evidence**	22	Present assessments of certainty (or confidence) in the body of evidence for each outcome assessed.	None
**DISCUSSION**			
**Discussion**	23a	Provide a general interpretation of the results in the context of other evidence.	Line 485-498

Topic	No.	Item	Location where item is reported
	23b	Discuss any limitations of the evidence included in the review.	Line 972-987
	23c	Discuss any limitations of the review processes used.	Line 979-983
	23d	Discuss implications of the results for practice, policy, and future research.	Line 913-930
**OTHER INFORMATION**			
**Registration and protocol**	24a	Provide registration information for the review, including register name and registration number, or state that the review was not registered.	N/A
	24b	Indicate where the review protocol can be accessed, or state that a protocol was not prepared.	Protocol not available
	24c	Describe and explain any amendments to information provided at registration or in the protocol.	N/A
**Support**	25	Describe sources of financial or non-financial support for the review, and the role of the funders or sponsors in the review.	Line 1007
**Competing interests**	26	Declare any competing interests of review authors.	Line 1018
**Availability of data, code and other materials**	27	Report which of the following are publicly available and where they can be found: template data collection forms; data extracted from included studies; data used for all analyses; analytic code; any other materials used in the review.	All data used in Tables

PRIMSA Abstract Checklist

Topic	No.	Item	Reported?
**TITLE**			
**Title**	1	Identify the report as a systematic review.	Yes
**BACKGROUND**			
**Objectives**	2	Provide an explicit statement of the main objective(s) or question(s) the review addresses.	Yes
**METHODS**			

Topic	No.	Item	Reported?
**Eligibility criteria**	3	Specify the inclusion and exclusion criteria for the review.	Yes
**Information sources**	4	Specify the information sources (e.g. databases, registers) used to identify studies and the date when each was last searched.	Yes
**Risk of bias**	5	Specify the methods used to assess risk of bias in the included studies.	Yes
**Synthesis of results**	6	Specify the methods used to present and synthesize results.	Yes
**RESULTS**			
**Included studies**	7	Give the total number of included studies and participants and summarise relevant characteristics of studies.	Yes
**Synthesis of results**	8	Present results for main outcomes, preferably indicating the number of included studies and participants for each. If meta-analysis was done, report the summary estimate and confidence/credible interval. If comparing groups, indicate the direction of the effect (i.e. which group is favoured).	Yes
**DISCUSSION**			
**Limitations of evidence**	9	Provide a brief summary of the limitations of the evidence included in the review (e.g. study risk of bias, inconsistency and imprecision).	Yes
**Interpretation**	10	Provide a general interpretation of the results and important implications.	Yes
**OTHER**			
**Funding**	11	Specify the primary source of funding for the review.	N/A
**Registration**	12	Provide the register name and registration number.	N/A

From: Page MJ, McKenzie JE, Bossuyt PM, Boutron I, Hoffmann TC, Mulrow CD, et al. The PRISMA 2020 statement: an updated guideline for reporting systematic reviews. MetaArXiv. 2020, September 14. DOI: 10.31222/osf.io/v7gm2. For more information, visit: www.prisma-statement.org

Table S2

Search Strategy of Databases:

Embase Classic+Embase <1990 to 2023 Week 20>

#	Query	Results from 26 May 2023
1	(Clozapine or Clopine or Clopine 25 or Clorazil or Clorazil 25 or Clozitor or Leponex or Zaponex or Denzapine or dibenzodiazepine or (antipsychotic* or anti-psychotic* or neuroleptic or tranquili* or major tranquili*)).mp. [mp=title, abstract, heading word, drug trade name, original title, device manufacturer, drug manufacturer, device trade name, keyword heading word, floating subheading word, candidate term word]	183,135
2	limit 1 to (human and english language and yr="1990 -Current")	58,668
3	(pneumonia or pneumonias or experimental lung inflammation* or lung inflammation* or pneumonitis or pneumotides or pneumocystis or pneumocytes or bacterial pneumonia* or bronchopneumonia* or pneumococcal disease or pneumococcus or lobar pneumonia or Chest infection or acute chest syndrome or acid aspiration syndrome or Mendelson syndrome).mp.	491,491

	[mp=title, abstract, heading word, drug trade name, original title, device manufacturer, drug manufacturer, device trade name, keyword heading word, floating subheading word, candidate term word]	
4	limit 3 to (human and english language and yr="1990 -Current")	231,021
5	(Pneumococcal vaccine or pneumococcal vaccination or pneumonia vaccine or PCV10 or 10vPCV or PCV13 or PCV15 or PCV20 or PCV23 or 13vPCV or 15vPCV or 20vPCV or 23vPPV or Pneumococcal conjugate vaccines or Pneumococcal polysaccharide vaccine or Prevnar 13 or Vaxneuvance or Prevnar 20 or Pneumovax23).mp. [mp=title, abstract, heading word, drug trade name, original title, device manufacturer, drug manufacturer, device trade name, keyword heading word, floating subheading word, candidate term word]	11,820
6	limit 5 to (human and english language and yr="1990 -Current")	6,371
7	(schizophrenia* or treatment-resistant schizophrenia or Treatment resistant schizophrenia or resistant schizophrenia or schizophrenic disorder or schizophrenic disorders or dementia Praecox or hebephrenic or hebephrenic schizophrenia* or disorganised schizophrenia* or catatonic or catatonic schizophrenia* or treatment-refractory schizophrenia or treatment refractory schizophrenia* or refractory schizophrenia or schizophrenia* or paranoid schizophrenia or Pseudo psychopathic Schizophrenia* or Pseudopsychopathic Schizophrenia* or serious mental illness or schizoaffective or schizophreniform).mp.	259,890

	[mp=title, abstract, heading word, drug trade name, original title, device manufacturer, drug manufacturer, device trade name, keyword heading word, floating subheading word, candidate term word]	
8	limit 7 to (human and english language and yr="1990 -Current")	98,273
9	2 or 8	131,811
10	4 and 9	1,337
11	5 and 9	6

Database:

APA PsycArticles Full Text

#	Query	Results from 26 May 2023
1	(Clozapine or Clopine or Clopine 25 or Clorazil or Clorazil 25 or Clozitor or Leponex or Zaponex or Denzapine or dibenzodiazepine or (antipsychotic* or anti-psychotic* or neuroleptic or tranquili* or major tranquili*)).mp. [mp=title, abstract, full text, caption text]	3,414
2	limit 1 to yr="1990 -Current"	840
3	(schizophrenia* or treatment-resistant schizophrenia or Treatment resistant schizophrenia or resistant schizophrenia or schizophrenic disorder or schizophrenic disorders or dementia Praecox or hebephrenic or hebephrenic schizophrenia* or disorganised schizophrenia* or catatonic or catatonic schizophrenia* or treatment-refractory schizophrenia or treatment refractory schizophrenia* or refractory schizophrenia or schizophrenia* or paranoid schizophrenia or Pseudo psychopathic Schizophrenia* or Pseudopsychopathic Schizophrenia* or serious mental illness or schizoaffective or schizophreniform).mp. [mp=title, abstract, full text, caption text]	12,671

4	limit 3 to yr="1990 -Current"	3,894
5	(pneumonia or pneumonias or experimental lung inflammation* or lung inflammation* or pneumonitis or pneumotides or pneumocystis or pneumocytes or bacterial pneumonia* or bronchopneumonia* or pneumococcal disease or pneumococcus or lobar pneumonia or Chest infection or acute chest syndrome or acid aspiration syndrome or Mendelson syndrome).mp. [mp=title, abstract, full text, caption text]	402
6	limit 5 to yr="1990 -Current"	139
7	(Pneumococcal vaccine or pneumococcal vaccination or pneumonia vaccine or PCV10 or 10vPCV or PCV13 or PCV15 or PCV20 or PCV23 or 13vPCV or 15vPCV or 20vPCV or 23vPPV or Pneumococcal conjugate vaccines or Pneumococcal polysaccharide vaccine or Prevnar 13 or Vaxneuvance or Prevnar 20 or Pneumovax23).mp. [mp=title, abstract, full text, caption text]	3
8	limit 7 to yr="1990 -Current"	2
9	2 or 4	4,310
10	6 and 9	15
11	8 and 9	0

Database:

Ovid MEDLINE(R) ALL <1990 to May 25, 2023>

#	Query	Results from 26 May 2023
1	(Clozapine or Clopine or Clopine 25 or Clorazil or Clorazil 25 or Clozitor or Leponex or Zaponex or Denzapine or dibenzodiazepine or (antipsychotic* or anti-psychotic* or neuroleptic or tranquili* or major tranquili*)).mp. [mp=title, book title, abstract, original title, name of substance word, subject heading word, floating sub-heading word, keyword heading word, organism supplementary concept word, protocol supplementary concept word, rare disease supplementary concept word, unique identifier, synonyms, population supplementary concept word, anatomy supplementary concept word]	101,963
2	limit 1 to (english language and humans and yr="1990 -Current")	22,167
3	(pneumonia or pneumonias or experimental lung inflammation* or lung inflammation* or pneumonitis or pneumotides or pneumocystis or pneumocytes or bacterial pneumonia* or bronchopneumonia* or pneumococcal disease or pneumococcus or lobar pneumonia or Chest infection or acute chest syndrome or acid aspiration syndrome or Mendelson syndrome).mp. [mp=title, book title, abstract, original title, name of substance word, subject heading word, floating sub-heading word, keyword heading word, organism supplementary concept word, protocol supplementary concept word, rare disease	261,554

	supplementary concept word, unique identifier, synonyms, population supplementary concept word, anatomy supplementary concept word]	
4	limit 3 to (english language and humans and yr="1990 -Current")	95,622
5	(schizophrenia* or treatment-resistant schizophrenia or Treatment resistant schizophrenia or resistant schizophrenia or schizophrenic disorder or schizophrenic disorders or dementia Praecox or hebephrenic or hebephrenic schizophrenia* or disorganised schizophrenia* or catatonic or catatonic schizophrenia* or treatment-refractory schizophrenia or treatment refractory schizophrenia* or refractory schizophrenia or schizophrenia* or paranoid schizophrenia or Pseudo psychopathic Schizophrenia* or Pseudopsychopathic Schizophrenia* or serious mental illness or schizoaffective or schizophreniform).mp. [mp=title, book title, abstract, original title, name of substance word, subject heading word, floating sub-heading word, keyword heading word, organism supplementary concept word, protocol supplementary concept word, rare disease supplementary concept word, unique identifier, synonyms, population supplementary concept word, anatomy supplementary concept word]	165,278
6	limit 5 to (english language and humans and yr="1990 -Current")	43,175
7	(Pneumococcal vaccine or pneumococcal vaccination or pneumonia vaccine or PCV10 or 10vPCV or PCV13 or PCV15 or PCV20 or PCV23 or 13vPCV or 15vPCV or 20vPCV or 23vPPV or Pneumococcal conjugate vaccines or Pneumococcal	8,279

	polysaccharide vaccine or Prevnar 13 or Vaxneuvance or Prevnar 20 or Pneumovax23).mp. [mp=title, book title, abstract, original title, name of substance word, subject heading word, floating sub-heading word, keyword heading word, organism supplementary concept word, protocol supplementary concept word, rare disease supplementary concept word, unique identifier, synonyms, population supplementary concept word, anatomy supplementary concept word]	
8	limit 7 to (english language and humans and yr="1990 -Current")	3,647
9	2 or 6	55,211
10	4 and 9	280
11	8 and 9	2

Database:

APA PsycInfo <1990 to May Week 4 2023>

#	Query	Results from 27 May 2023
1	(Clozapine or Clopine or Clopine 25 or Clorazil or Clorazil 25 or Clozitor or Leponex or Zaponex or Denzapine or dibenzodiazepine or (antipsychotic* or anti-psychotic* or neuroleptic or tranquili* or major tranquili*)).mp. [mp=title, abstract, heading word, table of contents, key concepts, original title, tests & measures, mesh word]	56,948
2	limit 1 to (human and english language and yr="1990 -Current")	13,788
3	(schizophrenia* or treatment-resistant schizophrenia or Treatment resistant schizophrenia or resistant schizophrenia or schizophrenic disorder or schizophrenic disorders or dementia Praecox or hebephrenic or hebephrenic schizophrenia* or disorganised schizophrenia* or catatonic or catatonic schizophrenia* or treatment-refractory schizophrenia or treatment refractory schizophrenia* or refractory schizophrenia or schizophrenia* or paranoid schizophrenia or Pseudo psychopathic Schizophrenia* or Pseudopsychopathic Schizophrenia* or serious mental illness or schizoaffective or schizophreniform).mp. [mp=title, abstract, heading word, table of contents, key concepts, original title, tests & measures, mesh word]	154,489

4	limit 3 to (human and english language and yr="1990 -Current")	42,061
5	(pneumonia or pneumonias or experimental lung inflammation* or lung inflammation* or pneumonitis or pneumotides or pneumocystis or pneumocytes or bacterial pneumonia* or bronchopneumonia* or pneumococcal disease or pneumococcus or lobar pneumonia or Chest infection or acute chest syndrome or acid aspiration syndrome or Mendelson syndrome).mp. [mp=title, abstract, heading word, table of contents, key concepts, original title, tests & measures, mesh word]	4,455
6	limit 5 to (human and english language and yr="1990 -Current")	2,756
7	(Pneumococcal vaccine or pneumococcal vaccination or pneumonia vaccine or PCV10 or 10vPCV or PCV13 or PCV15 or PCV20 or PCV23 or 13vPCV or 15vPCV or 20vPCV or 23vPPV or Pneumococcal conjugate vaccines or Pneumococcal polysaccharide vaccines or Prevnar 13 or Vaxneuvance or Prevnar 20 or Pneumovax23).mp. [mp=title, abstract, heading word, table of contents, key concepts, original title, tests & measures, mesh word]	147
8	limit 7 to (full text and english language and yr="1990 -Current")	9
9	2 or 4	48,306
10	6 and 9	175
11	8 and 9	0

Database:

PubMed Search History

Search number	Query		Filters	Results	Time
4	(Clozapine OR Clopine OR Clopine 25 OR Clorazil OR Clorazil 25 OR Clozitor OR Leponex OR Zaponex OR Denzapine OR dibenzodiazepine) OR (antipsychotic* OR anti-psychotic* OR neuroleptic OR tranquili* OR major tranquili*) OR (schizophrenia* OR treatment-resistant schizophrenia OR Treatment resistant schizophrenia OR resistant schizophrenia OR schizophrenic disorder OR schizophrenic disorders OR dementia Praecox OR hebephrenic OR hebephrenic schizophrenia* OR disorganised schizophrenia* OR catatonic OR catatonic schizophrenia* OR treatment-refractory schizophrenia OR treatment refractory schizophrenia* OR refractory schizophrenia OR schizophrenia* OR paranoid schizophrenia OR Pseudo psychopathic Schizophrenia* OR Pseudopsychopathic Schizophrenia* OR serious mental illness OR schizoaffective OR schizophreniform) AND (Pneumococcal vaccine OR pneumococcal vaccination OR pneumonia vaccine OR PCV10		Humans, English, from 2013 - 2023	97	18:20:23

	OR 10vPCV OR PCV13 OR PCV15 OR PCV20 OR PCV23 OR 13vPCV OR 15vPCV OR 20vPCV OR 23vPPV OR Pneumococcal conjugate vaccines OR Pneumococcal polysaccharide vaccine OR Prevnar 13 OR Vaxneuvance OR Prevnar 20 OR Pneumovax23)				
3	(Clozapine OR Clopine OR Clopine 25 OR Clorazil OR Clorazil 25 OR Clozitor OR Leponex OR Zaponex OR Denzapine OR dibenzodiazepine) OR (antipsychotic* OR anti-psychotic* OR neuroleptic OR tranquili* OR major tranquili*) OR (schizophrenia* OR treatment-resistant schizophrenia OR Treatment resistant schizophrenia OR resistant schizophrenia OR schizophrenic disorder OR schizophrenic disorders OR dementia Praecox OR hebephrenic OR hebephrenic schizophrenia* OR disorganised schizophrenia* OR catatonic OR catatonic schizophrenia* OR treatment-refractory schizophrenia OR treatment refractory schizophrenia* OR refractory schizophrenia OR schizophrenia* OR paranoid schizophrenia OR Pseudo psychopathic Schizophrenia* OR Pseudopsychopathic Schizophrenia* OR serious mental illness OR schizoaffective OR schizophreniform) AND (Pneumococcal vaccine OR pneumococcal vaccination OR pneumonia vaccine OR PCV10 OR 10vPCV OR PCV13 OR PCV15 OR PCV20 OR PCV23 OR 13vPCV OR 15vPCV OR		Humans, English	105	18:20:18

	20vPCV OR 23vPPV OR Pneumococcal conjugate vaccines OR Pneumococcal polysaccharide vaccine OR Prevnar 13 OR Vaxneuvance OR Prevnar 20 OR Pneumovax23)				
2	(Clozapine OR Clopine OR Clopine 25 OR Clorazil OR Clorazil 25 OR Clozitor OR Leponex OR Zaponex OR Denzapine OR dibenzodiazepine) OR (antipsychotic* OR anti-psychotic* OR neuroleptic OR tranquili* OR major tranquili*) OR (schizophrenia* OR treatment-resistant schizophrenia OR Treatment resistant schizophrenia OR resistant schizophrenia OR schizophrenic disorder OR schizophrenic disorders OR dementia Praecox OR hebephrenic OR hebephrenic schizophrenia* OR disorganised schizophrenia* OR catatonic OR catatonic schizophrenia* OR treatment-refractory schizophrenia OR treatment refractory schizophrenia* OR refractory schizophrenia OR schizophrenia* OR paranoid schizophrenia OR Pseudo psychopathic Schizophrenia* OR Pseudopsychopathic Schizophrenia* OR serious mental illness OR schizoaffective OR schizophreniform) AND (pneumonia OR pneumonias OR experimental lung inflammation* OR lung inflammation* OR pneumonitis OR pneumotides OR pneumocystis OR pneumocytes OR bacterial pneumonia* OR bronchopneumonia* OR pneumococcal disease OR pneumococcus OR		Humans, English, from 2013 - 2023	1,392	18:19:27

	lobar pneumonia OR Chest infection OR acute chest syndrome OR acid aspiration syndrome OR Mendelson syndrome)				
1	(Clozapine OR Clopine OR Clopine 25 OR Clorazil OR Clorazil 25 OR Clozitor OR Leponex OR Zaponex OR Denzapine OR dibenzodiazepine) OR (antipsychotic* OR anti-psychotic* OR neuroleptic OR tranquili* OR major tranquili*) OR (schizophrenia* OR treatment-resistant schizophrenia OR Treatment resistant schizophrenia OR resistant schizophrenia OR schizophrenic disorder OR schizophrenic disorders OR dementia Praecox OR hebephrenic OR hebephrenic schizophrenia* OR disorganised schizophrenia* OR catatonic OR catatonic schizophrenia* OR treatment-refractory schizophrenia OR treatment refractory schizophrenia* OR refractory schizophrenia OR schizophrenia* OR paranoid schizophrenia OR Pseudo psychopathic Schizophrenia* OR Pseudopsychopathic Schizophrenia* OR serious mental illness OR schizoaffective OR schizophreniform) AND (pneumonia OR pneumonias OR experimental lung inflammation* OR lung inflammation* OR pneumonitis OR pneumotides OR pneumocystis OR pneumocytes OR bacterial pneumonia* OR bronchopneumonia* OR pneumococcal disease OR pneumococcus OR		Humans, English	1,830	18:19:17

	lobar pneumonia OR Chest infection OR acute chest syndrome OR acid aspiration syndrome OR Mendelson syndrome)				

Database:

SCOPUS search terms

ID	Name	Query	Documents
#3	pneumon clozapine 1989 2024	TITLE-ABS-KEY (pneumon* AND clozapine) AND PUBYEAR > 1989 AND PUBYEAR < 2024	487

Vaccination Search

# ▲	Searches	Results
1	(Clozapine or Clopine or Clozaril or Clozitor or Leponex or Zaponex or Denzapine or dibenzodiazepine).mp. [mp=ti, ot, ab, tx, ct, sh, kw, fx, hw, sw, tn, dm, mf, dv, kf, dq, bt, nm, ox, px, rx, an, ui, sy, ux, mx, tc, id, tm]	82457
2	limit 1 to english language [Limit not valid in Your Journals@Ovid,DARE,CDSR,ACP Journal Club,CCA,CLCMR,Journals@Ovid,JBI EBP Database; records were retained]	77012
3	limit 2 to full text [Limit not valid in Your Journals@Ovid; records were retained , Limit not valid in DARE,CDSR,ACP Journal Club,CCA,CLCMR,CLHTA,CLEED; records were eliminated]	16499
4		12796

	limit 3 to yr="2000 - 2023" [Limit not valid in DARE; records were retained]	
5	(Severe mental illness or serious mental illness or enduring mental illness or schizophrenia or schizophrenic or bipolar).mp. [mp=ti, ot, ab, tx, ct, sh, kw, fx, hw, sw, tn, dm, mf, dv, kf, dq, bt, nm, ox, px, rx, an, ui, sy, ux, mx, tc, id, tm]	1063467
6	limit 5 to english language [Limit not valid in Your Journals@Ovid,DARE,CDSR,ACP Journal Club,CCA,CLCMR,Journals@Ovid,JBI EBP Database; records were retained]	979050
7	limit 6 to full text [Limit not valid in Your Journals@Ovid; records were retained , Limit not valid in DARE,CDSR,ACP Journal Club,CCA,CLCMR,CLHTA,CLEED; records were eliminated]	210973
8	limit 7 to yr="2000 - 2023" [Limit not valid in DARE; records were retained]	146548
9	(vaccination or vaccine or immunisation or immunization or vaccines).mp. [mp=ti, ot, ab, tx, ct, sh, kw, fx, hw, sw, tn, dm, mf, dv, kf, dq, bt, nm, ox, px, rx, an, ui, sy, ux, mx, tc, id, tm]	1640401
10	limit 9 to english language [Limit not valid in Your Journals@Ovid,DARE,CDSR,ACP Journal Club,CCA,CLCMR,Journals@Ovid,JBI EBP Database; records were retained]	1474093
11	limit 10 to full text [Limit not valid in Your Journals@Ovid; records were retained , Limit not valid in DARE,CDSR,ACP Journal Club,CCA,CLCMR,CLHTA,CLEED; records were eliminated]	286313
12	limit 11 to yr="2000 - 2023" [Limit not valid in DARE; records were retained]	206789
13	4 or 8	150587

14	12 and 13	1069
	remove duplicates from 14	895
	University of Melbourne Full Text Journals	818
	EBM Reviews - Database of Abstracts of Reviews of Effects <1st Quarter 2016>	0
	EBM Reviews - Cochrane Database of Systematic Reviews <2005 to November 20, 2023>	0
	EBM Reviews - ACP Journal Club <1991 to October 2023>	0
	EBM Reviews - Cochrane Clinical Answers <November 2023>	0
	EBM Reviews - Cochrane Central Register of Controlled Trials <October 2023>	0
	EBM Reviews - Cochrane Methodology Register <3rd Quarter 2012>	0
	EBM Reviews - Health Technology Assessment <4th Quarter 2016>	0
	EBM Reviews - NHS Economic Evaluation Database <1st Quarter 2016>	0
	APA PsycArticles Full Text	2
	JBI EBP Database <Current to November 13, 2023>	4
	Embase Classic+Embase <1947 to 2023 November 25>	56
	Ovid Emcare <1995 to 2023 Week 46>	6
	Ovid MEDLINE(R) ALL <1946 to November 25, 2023>	5
15	APA PsycInfo <1806 to November 2023 Week 3>	4
